# Synergistic Effects of Multiple Environmental Factors on Degradation of Hydrogenated Nitrile Rubber Seals

**DOI:** 10.3390/polym10080897

**Published:** 2018-08-10

**Authors:** Weitao Lou, Weifang Zhang, Tingzhu Jin, Xuerong Liu, Wei Dai

**Affiliations:** School of Reliability and Systems engineering, Beihang University, Beijing 100191, China; louweitao@buaa.edu.cn (W.L.); zhangweifang@buaa.edu.cn (W.Z.); jingtingzhu@buaa.edu.cn (T.J.); liuxuerong@buaa.edu.cn (X.L.)

**Keywords:** hydrogenated nitrile rubber seals, degradation, compressive stress, elevated temperature, hydraulic oil, crosslinking

## Abstract

Degradation tests of hydrogenated nitrile rubber seals, often used as sealing components in hydraulic systems, were conducted under the free and compression state in air and hydraulic oil at three elevated temperatures for several days to investigate the synergistic effects among three factors. The crosslinking and chain scission reactions both occurred simultaneously at higher temperature during the degradation process, and crosslinking predominated for most cases. Additionally, the synergistic effect between compression stress and hydraulic oil further slowed the degradation rate by limiting oxygen access. However, the higher temperature and hydraulic oil both promoted the formation of oxidation products, whereas the compression stress restrained the formation of amide groups. The fracture morphology results show that the defects gradually formed on the fracture surface, especially for the uncompressed specimens. The increase of the compression set aged in air was more than that in hydraulic oil, implying the more serious degradation. Moreover, rubber seals under the synthetic effect of three environmental factors presented the minimum degradation level. The degradation of the compressed and uncompressed specimens exposed to hydraulic oil is more serious than that of specimens exposed to air.

## 1. Introduction

Hydrogenated nitrile butadiene rubbers are frequently used in aerospace, automobile manufacturing, and petroleum industries under different environmental conditions, due to its excellent physical and mechanical properties, and good resistance to chemical and thermal degradation [1,2]. For example, in hydraulic sealing fields, hydrogenated nitrile butadiene rubber is often applied to inhibit leakage of gases or/and liquids by using rubber seal components such as O–rings and gaskets [3]. However, under practical operating environments, the seals are often subjected to the single or synergistic effect of the various kinds of environmental factors such as elevated temperature, oxygen, chemical medium, and mechanical load stress, etc. [4,5,6,7,8]. Under the long-term influence of these environmental factors, the rubber seals gradually degrade and eventually bring about the failure of seals [9,10]. Thus, assessing the influence of environment factors on the degradation process of rubber material plays a prominent part in its practical use.

Various exposure conditions lead to degradation of rubber seals, where oxygen and temperature are significant aging factors [11,12]. Thermal degradation behavior research of nitrile butadiene rubber in hot air has been studied for many decades [13,14,15,16,17]. The degradation mainly includes physical aging and chemical aging. Physical aging is primarily due to the volatilization and loss of fillers or/and additives, and the rearrangement and disentanglement of molecular chains [18,19]. In addition, loss of antioxidants may accelerate the degradation process of rubber seals with little or no protection during subsequent thermal oxidation [5]. Chemical aging consists of various oxidation reactions including crosslinking and chain scission, etc. [20]. The changes of network structure are mainly attributed to the competition between crosslinking and chain scission, and excessive crosslinking may result in a denser network structure, which has a negative effect on the mechanical properties of rubber materials [21,22].

When rubber seals are used in the hydraulic system, they unavoidably are exposed to hydraulic oil. During the degradation process, hydraulic oil gradually diffuses into the interior of seals, leading to swelling of the rubber network structure [1,3]. Additionally, hydraulic oil could dissolve or extract soluble components (e.g., antioxidants) from the rubber during the exposure process, which result in poor oxidation resistance and lower mechanical properties of rubber materials [5,23,24]. The compatibility between rubber matrix and fillers also becomes worse [25,26,27]. Besides, the oil type has a direct effect on degradation processes of rubber materials [23,25,28]. Biodiesel consisting of fatty acid esters has a more excellent dissolving capacity than diesel fuel, which is a mixture of saturated alkanes, according to the principle of “like dissolves like” [23,29]. However, under harsh environmental conditions, the oxidation of biodiesel is more likely to happen and form some oxidation products such as aldehydes, ketones, and carboxylate acids [26,30]. The oxidation products of biodiesel could result in oxidative crosslinking in the matrix structures of rubber materials by further promoting the formation of carbonyl and etheric groups [27,31]. Moreover, the addition of multiwall carbon nanotubes to the rubber matrix could increase its crystallinity. The crystalline regions exert motion restrictions to large segments of the macromolecules in the amorphous phase and to polar parts of the systems, and further improves thermal resistance, chemical resistance, and environmental stability of rubber materials during the degradation process [32].

Under actual working conditions, to obtain excellent seal effect, the rubber seals are often subjected to compression stress (constant deformation) and maintained in this state for a long time [7]. The compression stress of the seals tends to decrease with exposure time, which is attributed to stress relaxation and oxidation reactions [20,33]. Both the physical and chemical process have a negative effect on dynamic performances of rubber seals, which markedly shortens the service life of seals and even leads to leakage of gases or oils [34]. Furthermore, the compression stress could restrain oxygen access and inhibit thermo-oxidative aging. Nevertheless, studies of rubber seals subjected to compression stress are relatively rare, despite the fact that the seals often undergo pre-set deformation.

Many researchers have focused on degradation behavior and mechanism of various nitrile butadiene rubbers at high temperature, in chemical medium or under stress (tension or compression). However, in the case of hydraulic systems, rubber O-rings are subjected to more severe and complicated working conditions, such as elevated temperature, compression stress, and hydraulic oil, resulting in an extremely complicated processes of degradation of rubber seals [35,36]. This is mainly due to the synergistic effects of elevated temperature, liquid environment, and mechanical stress, especially for the synergistic effects between elevated temperature and other environment factors. Moreover, the synergistic effects of two and/or three environmental factors on degradation of rubber materials may be more or less serious than the single and/or combined factors, because, on the one hand, compression stress and hydraulic oil could limit oxygen access in rubber seals and slow thermal degradation process; on the other hand, the stress can bring about physical and chemical changes in rubber seals, such as compression stress relaxation, stress-induced chain scission, and rearrangement of rubber chains, which have a significant effect on degradation behavior of rubber seals [33]. For example, Zeng et al. [35] reported that the corrosion of samples under compression was more serious than that under the free state. Besides, corrosion without stress in liquid environments was more serious. Alcock et al. [36] investigated the changes in mechanical properties of hydrogenated nitrile butadiene rubber under simulated sweet oil exposure at high temperature and pressure. Guo et al. [37] studied the effect of the aging time on the thermal oil aging behavior of hydrogenated nitrile butadiene rubber (HNBR)/multi-walled carbon nanotube composites under free or compression states. Thus, synergistic effects of elevated temperature, hydraulic oil, and compressive stress on degradation of hydrogenated nitrile rubber seals still need to be investigated further.

In our previous paper, special attention was given to the effect of hydraulic oil on degradation behaviors of rubber seals at elevated temperature [38]. However, we still do not know what roles the synergistic effects of two and three environmental factors play in degradation of rubber seals under actual service conditions. Thus, on the existing basis of theoretical and experimental data, we further designed and conducted a series of systematic degradation experiments under the double and three environmental factors to obtain more data. Additionally, part of the data such as the crosslinking density, the fourier transform infrared spectroscopy (FTIR), and the mechanical properties has been presented in a previous paper [38] to compare to the half of the data which appears to be new. The aim is to understand better the synergistic effects of elevated temperature, hydraulic oil, and compressive stress on degradation behavior and mechanisms of hydrogenated nitrile rubber O-rings. During the degradation tests, the rubber specimens under compression or not under compression were exposed to air and hydraulic oil at three elevated temperatures for up to 64 days. The constant compression deformation was applied by self-designed compression apparatus. Then, the chemical changes in the aged specimens were investigated by attenuated total reflection-Fourier transform infrared spectroscopy (ATR-FTIR) and solvent swelling test. The physical and mechanical properties of the aged specimens were studied by compression set, and mechanical properties. The tensile fracture morphologies of rubber specimens were assessed by scanning electron microscopy (SEM).

## 2. Materials and Methods

### 2.1. Material and Hydraulic Oil

The vulcanized hydrogenated nitrile butadiene rubber O-rings with 35% acrylonitrile content provided by Changsha 5712 Aircraft Industry Corporation, Ltd., (Changsha China) were used to conduct the accelerated aging test. In addition to hydrogenated nitrile rubber, zinc oxide, sulfur, carbon black, plasticizer, stearic acid, and antioxidant were also included in the composition of the rubber sheets. The formulation is shown in Table 1. The material was compounded by using a two-roll mill at the constant temperature of 151 °C and pressure of 5.88 to 11.76 MPa for 40 min. Then, the materials were manufactured into O-rings that measured Φ17 mm × 6 mm (inner diameter × cross section diameter). In addition, sheets of 2 mm thickness were prepared for mechanical properties and crosslinking density measurements.

The hydraulic oil is 46# aviation hydraulic oil, which was supplied by Great Wall Lube Oil (SINOPEC, Beijing, China. The main components of the hydraulic oil were mineral oils, antioxidant 264, tricresyl phosphate, lanolin, dibutyl phosphite, N, and N-din-butyl-diethylaminomethylene-benzotriazole. The density of the hydraulic oil was 0.862 kg/L at 25 °C. The kinematic viscosity was 38.12 mm^2^/s at 50 °C. The flash temperature was 165 °C. The acid value was 0.78 mg KOH/g.

### 2.2. Aging Methods

**Figure 1:**
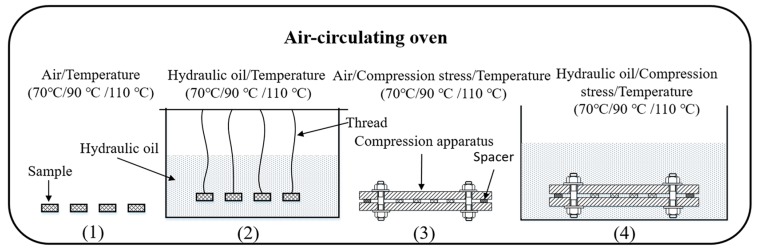
Schematic diagram of rubber specimens under different aging conditions.

In order to investigate synergistic effects of elevated temperature, hydraulic oil and compressive stress on degradation behavior, and the mechanism of degradation of hydrogenated nitrile butadiene rubber O-rings under actual working conditions, degradation tests under different conditions were conducted in air-circulating ovens. For compressed specimens, the specimens including O-rings and strips were placed in self-designed compression simulators, and the specimens were compressed to 30% of their original height based on the actual compression state. To keep the constant compression set, spacers were designed and placed in between two steel plates, and the dimension of the spacer was Φ10 mm × 4.2 mm (cross section diameter × height). Afterwards, the uncompressed and compressed specimens were exposed to air or immersed in hydraulic oil. Then, the specimens were put in the air-circulating ovens at selected temperatures. The schematic diagram of rubber specimens under different aging conditions are shown in Figure 1. The test temperatures were selected at 70 °C, 90 °C, and 110 °C and examined after 2, 4, 8, 16, 32, and 64 days. Additionally, three parallel specimens were prepared for degradation tests under different aging conditions, respectively. After the oil exposure tests, the aged specimens were removed from the containers at selected days, and the remaining oil solution on the specimen surface was cleaned with filter paper.

### 2.3. Characterization Methods

#### 2.3.1. Crosslinking Density

The equilibrium swelling behaviors of the aged specimens were investigated to evaluate the changes in rubber network structure by the swelling tests. The specimens were cut from the aging strips. The length, width, and thickness of the specimens were 20 mm, 10 mm, and 2 mm. The tests were carried out in acetone at 23 °C for 96 h. Three parallel specimens were measured over time for each exposure environment, and the average values are shown in this paper. The Flory–Rehner equation was applied to calculate the crosslinking density of the three-dimensional network:

(1) $$V_{e}=-\frac{1}{V}\left[\frac{\ln(1-V_{r})+V_{r}+\mu V_{r}^{2}}{V_{r}^{1/3}-V_{r}/2}\right]$$

where $V_{e}$ was the crosslinking density, μ was the polymer-solvent interaction parameter, V was the molecular volume of acetone, and $V_{r}$ was the volume fraction of hydrogenated nitrile butadiene rubber after immersion in acetone [11,39].

#### 2.3.2. ATR-FTIR Analysis

Attenuated total reflection-Fourier transform infrared (ATR-FTIR, Thermo Nicolet 6700, Waltham, MA, USA) spectroscopy was applied to evaluate the chemical changes in surfaces of the specimens aged under different conditions. The range of spectra was 400–4000 cm^−1^ with the resolution of 4 cm^−1^. The dates measured at three different positions on the surfaces of the aged specimens were averaged in order to assess the peak identification more accurate. Additionally, the fresh hydraulic oil was also analyzed by ATR-FTIR spectroscopy.

#### 2.3.3. Scanning Electron Microscopy (SEM) Analysis

The tensile fracture morphologies of the samples before and after exposure to different conditions were analyzed using SEM (JSM-6480, JEOL, Beijing, China). All the samples after tensile tests were immediately placed in a sealed bag to inhibit the fracture surface from being polluted.

#### 2.3.4. Compression Set

Compression Set (CS) gives information about the resistance to deformation of the O-rings before and after exposure under compression. At selected days, the specimens were taken out and then recovered until no changes occurred in height at room temperature. It was calculated using the following formula:

(2) $$CS=\frac{h_{0}-h_{2}}{h_{0}-h_{1}}\times 100\%$$

where *h*_0_ was the initial height, *h*_1_ was the compressed height, and *h*_2_ was the measured recovered height.

#### 2.3.5. Tensile Properties

Mechanical properties of the aged specimens were evaluated by tensile tests. The tensile specimens were conducted on each group according to GB/T 5720:1993 (China) using a CMT5504 electronic tensile testing machine (MTS, Eden, MN, USA) at the strain rate of 50 mm/min. The geometry of the testing samples cut from the aging strips was dumbbell-type (length = 115 mm, width = 25 mm, thickness = 2 mm). Three samples were tested in order to get a reliable result. The Young’s modulus (secant modulus at 5% strain), tensile strength, and elongation at break of three samples were determined for each aged samples.

## 3. Results

### 3.1. Crosslinking Density

**Figure 2:**
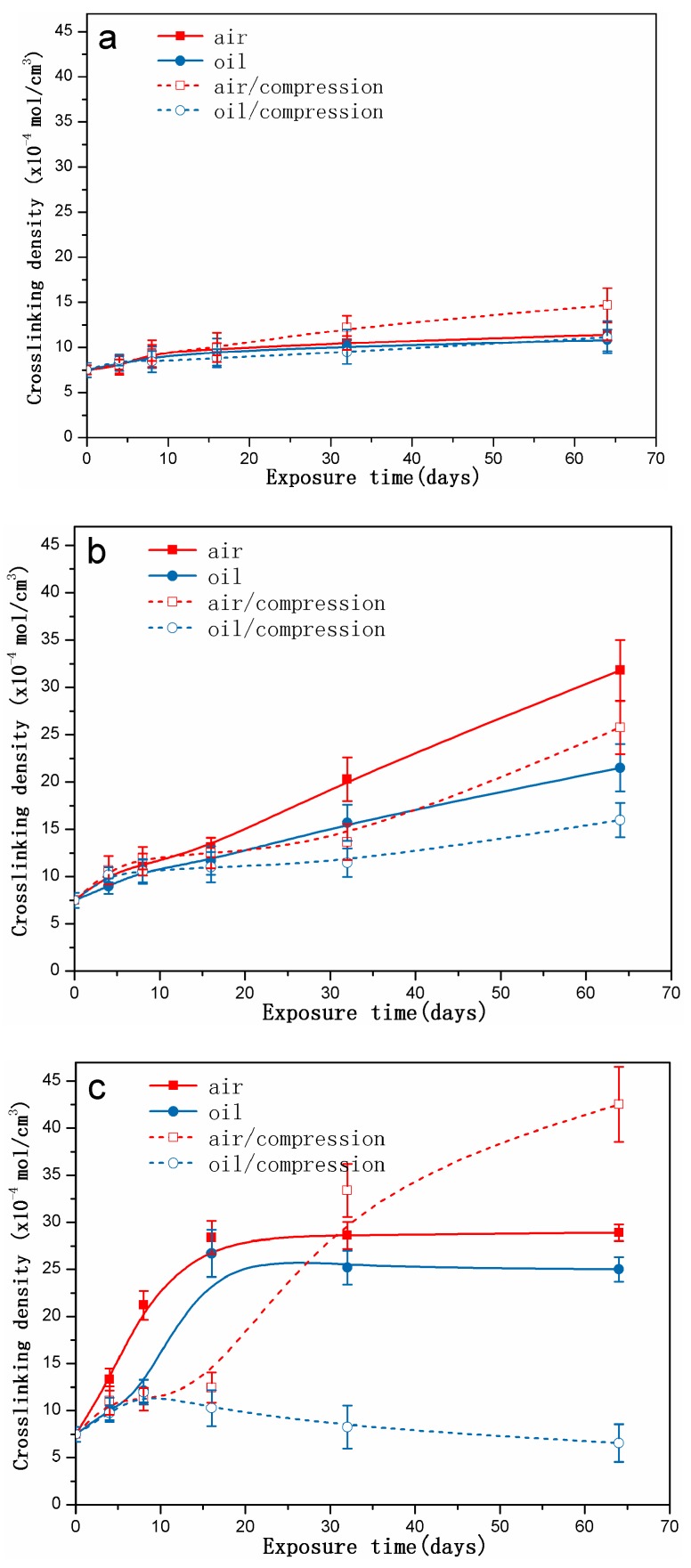
The changes in crosslinking density of rubber specimens exposed to different conditions as a function of exposure time: (**a**) at 70 °C; (**b**) at 90 °C; (**c**) at 110 °C.

Figure 2 shows the changes in crosslinking density of the rubber specimens exposed to different conditions as a function of exposure time at 70 °C, 90 °C, and 110 °C. Figure 2a shows a similar trend that the crosslinking density of all the rubber specimens increased slightly with increasing exposure time, in which the crosslinking density of the uncompressed specimens exposed to air show more increase compared to the other cases.

Figure 2b presents the changes in crosslinking density of the specimens before and after exposure to different conditions at 90 °C. The crosslinking density slightly increased in the first 16 days and then increased remarkably in the next days. Furthermore, the crosslinking density values for the uncompressed rubber specimens exposed to air was higher than that of the uncompressed specimens exposed to hydraulic oil, implying the more serious degradation due to oxidation. When the rubber specimens were subjected to the compressive stress, we found that the crosslinking density for the compressed specimens were lower than that under the free state, which indicates that the compressive stress could limit the oxidation process.

Figure 2c shows the changes in crosslinking density of the specimens before and after exposure to different conditions at 110 °C. We observed that the crosslinking density of the specimens exposed to air and hydraulic oil both increased sharply in the first 16 days, and then remained constant during the next days. Additionally, the crosslinking density exposure to air is greater than that exposure to hydraulic oil, implying the exhibition of hydraulic oil on the oxidation. However, for the compressed specimens, the crosslinking density changed significantly and differently, which demonstrated the noticeable effect of compression stress. The crosslinking density of the uncompressed specimens exposed to air showed a slight increase in the first eight days and then tended to increase markedly after that. Finally, a higher increase of value was observed as compared to that exposure to the other aging conditions. For the compressed specimens exposed to hydraulic oil at 110 °C, the crosslinking density increased first until reaching a maximum in the first eight days, and then decreased slightly. These results demonstrate that high temperature, hydraulic oil, and compressive stress all played an important role in the changes of crosslinking density, especially high temperature and compressive stress.

### 3.2. ATR-FTIR Analysis

**Figure 3:**
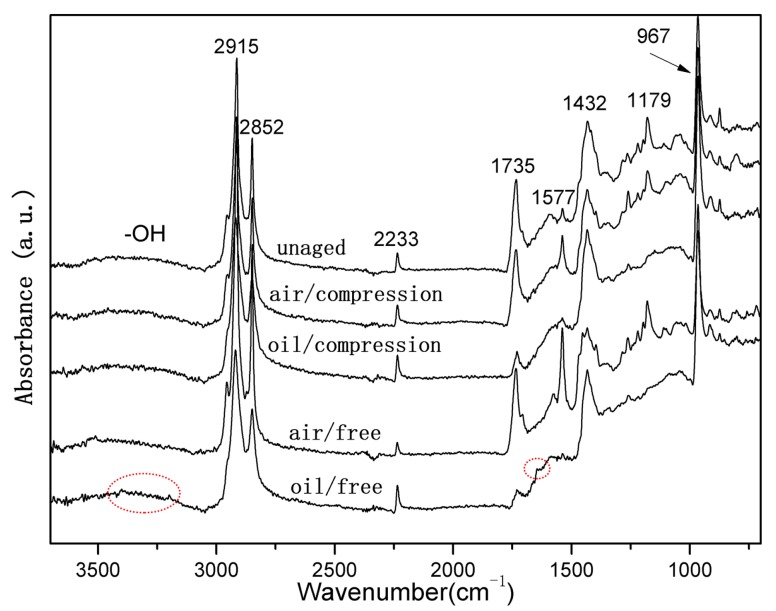
ATR-FTIR spectra of both uncompressed and compressed rubber seals before and after exposure to air and oil for 32 days at 70 °C.

**Figure 4:**
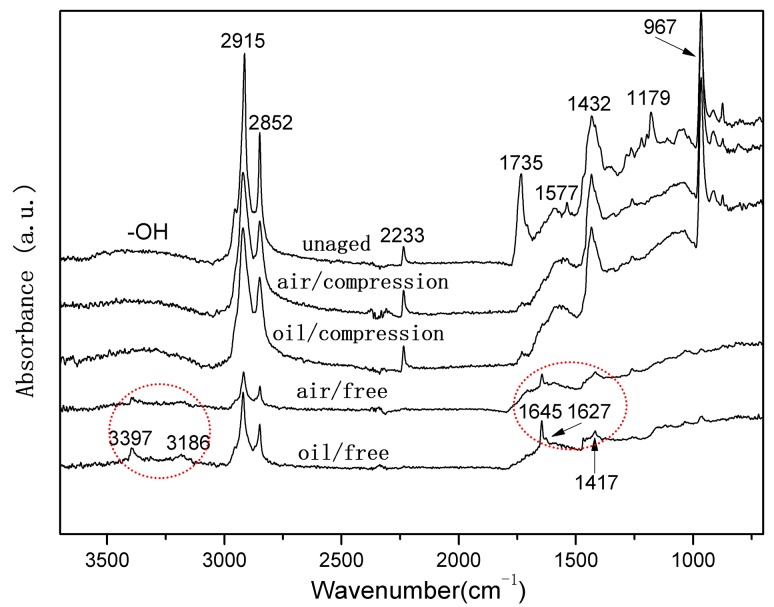
ATR-FTIR spectra of both uncompressed and compressed rubber seals before and after exposure to air and oil for 32 days at 110 °C.

In order to determine the chemical changes in the rubber network, the compressed and uncompressed specimens exposed to air and hydraulic oil at three elevated temperatures were analyzed via ATR-FTIR. Figure 3 and Figure 4 show ATR-FTIR spectra of both uncompressed and compressed rubber seals before and after exposure to air and oil for 32 days at 70 °C and 110 °C, respectively. The peak assignments for the ATR-FTIR spectra are shown in Table 2. In addition, the broad peak at 3365 cm^−1^ can be attributed to the O–H symmetric stretching vibration [20,40]. It can be seen that the new peak around 3365 cm^−1^ occurred and gradually strengthened with exposure time increasing, implying the formation of hydroxyl groups due to oxidation of the rubber seals. Additionally, the small peaks at 3394 cm^−1^ and 3185 cm^−1^ are assigned to the N–H antisymmetric stretching vibration and the N–H symmetric stretching vibration, respectively, and the new peaks at 1651 cm^−1^, 1627 cm^−1^, and 1417 cm^−1^ belong to C=O stretching vibration, the N–H deformation vibration and C–N stretching vibration, respectively, which demonstrate the formation of amide groups (R–CO–NH_2_) [10,40]. As shown in Figure 3 and Figure 4, we can observe that, for both the uncompressed specimens after exposure to hydraulic oil for 32 days at 70 °C and 110 °C, the nitrile groups reacted and were converted into amide groups, while the reactions were more sensitive to the higher temperature. Furthermore, it can be seen that for the uncompressed specimens after 32 days of exposure to air, the amide groups were generated at 110 °C, but not formed at 70 °C. By comparison the spectra between exposure to air and hydraulic oil, we found that hydraulic oil could accelerate the reactions of the nitrile groups, which were in agreement with the disappearance of the peak at 2233 cm^−1^. However, all the spectra of the compressed specimens exposed to air and hydraulic oil at 70 °C and 110 °C did not present these new peaks attributed to amide groups, which indicated that the reactions of the nitrile groups did not occur, implying inhibition for the reactions.

It also can be seen from Figure 3 and Figure 4, when the uncompressed rubber seals were exposed to air and hydraulic oil for 32 days at 110 °C, the peaks at 2915 cm^−1^ and 2852 cm^−1^ decreased obviously in intensity, and the decrease was more for the exposure to hydraulic oil. This is mainly attributed to the volatilization and migration of paraffin, due to the existence of elevated temperature and hydraulic oil [5,19,20]. In addition, the decrease in intensity of the peak at 1735 cm^−1^ was mostly due to the consumption of carbonyl groups, or attributed to the loss of plasticizer, which matches well with the decrease of the peaks at 1577 cm^−1^ and 1179 cm^−1^ [11,20]. The more these peaks decreased for the uncompressed specimens exposed to hydraulic oil at 110 °C implies that the elevated temperature and hydraulic oil promote the consumption of carbonyl groups, or the loss of plasticizer, but the compression stress limits these process. The remarkable decrease in intensity of the peak at 962 cm^−1^ for the uncompressed specimens after exposure to air and hydraulic oil for 32 days at 110 °C indicates a more serious degradation, compared to the other different aging conditions.

It can be concluded that elevated temperature and hydraulic oil have a significant effect on the surface chemical changes of rubber seals. The higher the aging temperature was, the more severe the oxidation was. The more serious degradation occurred for the specimens exposed to hydraulic oil, especially for the synergistic effect of the elevated temperature and hydraulic oil. However, we found that the compression stress obviously inhibited the migrations of additives and the reactions of chemical groups.

**Figure 5:**
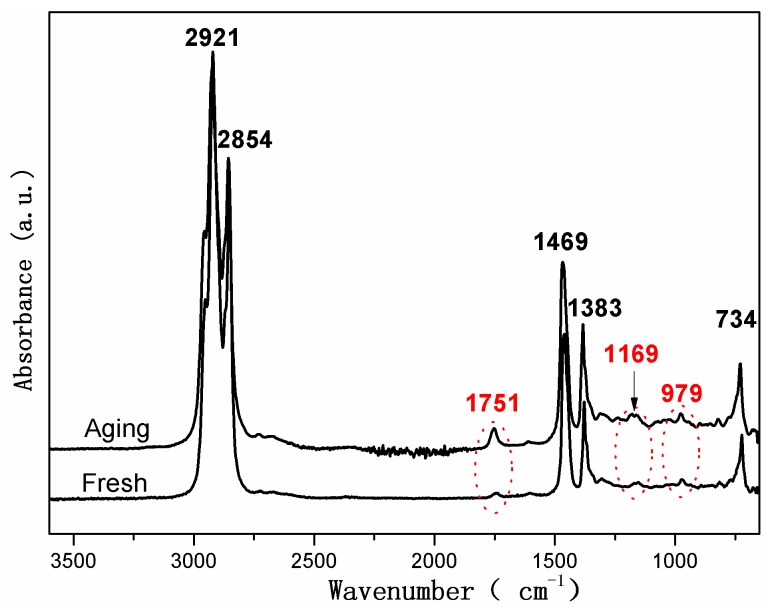
ATR-FTIR spectra of hydraulic oil before and after immersion at 110 °C.

Figure 5 shows the ATR-FTIR spectra of hydraulic oil before and after immersion at 110 °C. The peaks at 2921 cm^−1^ and 2854 cm^−1^ are attributed to symmetric and asymmetric stretching vibration of the CH_2_ groups, respectively. The peak at 1469 cm^−1^ is assigned to the bending vibrations of the CH_2_ and CH_3_ groups. The peaks at 1383 cm^−1^ and 734 cm^−1^ belong to the bending vibrations of CH_2_ groups and the overlapping of the CH_2_ rocking vibration, respectively [25]. The spectra confirms that the hydraulic oil mainly consisted of saturated hydrocarbons with different lengths of carbon chains. In addition, the weak peaks at 1751 cm^−1^ and 1169 cm^−1^ are attributed to the carbonyl ester functional group and stretching vibration of the C–O ester group, implying the presence of additives in fresh and aging oil. After exposure, the increases of the two absorption peaks were mainly due to the migration of additives or oxidation products of aging seals, which is in agreement with the analysis of the peak at 1735 cm^−1^ for the FTIR spectrum of the aging seals.

### 3.3. Fracture Morphology

**Figure 6:**
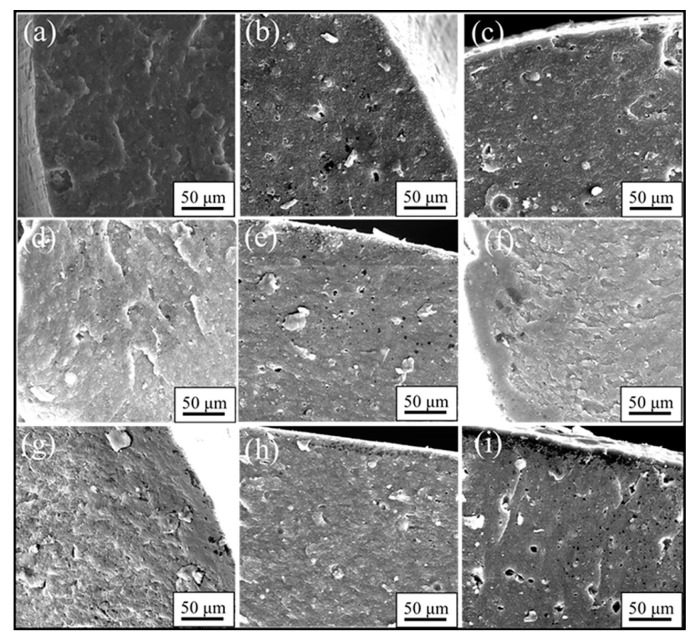
Tensile fracture surface of the rubber specimens before and after exposure to different aging conditions at 70 °C and 110 °C for 64 days: (**a**) unaged; (**b**) exposure to air at 70 °C; (**c**) exposure to hydraulic oil at 70 °C; (**d**) exposure to air at 70 °C under compression; (**e**) exposure to hydraulic oil at 70 °C under compression; (**f**) exposure to air at 110 °C; (**g**) exposure to hydraulic oil at 110 °C; (**h**) exposure to air at 110 °C under compression; (**i**) exposure to hydraulic oil at 110 °C under compression.

In order to further find the causes and process characteristics of rubber material degradation, fracture morphology analyses were performed using SEM. Figure 6 shows the fracture morphologies of the rubber specimens before and after exposure to different conditions at 70 °C and 110 °C for 64 days. It is obvious that the fillers and additives uniformly distributed in the rubber matrix for the unaged specimens and no obvious defects were observed (shown in Figure 6a). Nevertheless, for the specimens after 64 days of exposure at 70 °C, voids and particle agglomerates were gradually formed in the fracture surface, which became rough. Especially for the specimens immersed in hydraulic oil, the smaller and more voids appeared in the fracture surface. When the exposure temperature rose to 110 °C, we found that voids turned bigger and fracture surfaces became rougher. Additionally, a hardened brittle outer layer was formed at the edge region of the aged specimens, especially for the uncompressed specimens exposed to air and hydraulic oil, which was not obviously observed for the specimens aged at 70 °C. By comparing the fracture morphology under different aging conditions, it can be concluded that degradation of the specimens with or without stress immersed in hydraulic oil was more severely eroded as compared to that exposure to air, and the specimens exposed to air or hydraulic oil under uncompressed degraded more seriously than that under compressed.

### 3.4. Compression Set

**Figure 7:**
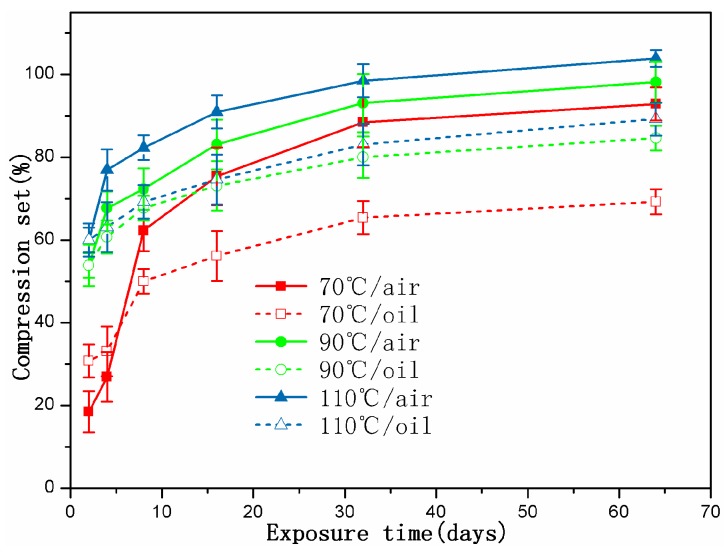
The changes in compression set of the compressed rubber seals exposed to different conditions as a function of exposure time.

Figure 7 shows the changes in compression set of the compressed rubber seals exposed to different conditions with exposure time. We observed that, for all the cases, the compression set showed a gradual increase with exposure time increasing. Moreover, the higher the temperature was, the greater the compression set was. Meanwhile, the compression set of the compressed rubber seals exposed to air was higher than that of the specimens exposed to hydraulic oil for the same aging conditions, implying the more serious degradation. For the rubber seals after 64 days of exposure to air, the value of the compression set even exceeded 100%, which indicates that severe shrinkage was formed in the rubber network structure, leading to the sealing failure of rubber seals. It could be concluded that hydraulic oil and temperature have a significant effect on the changes of compression set.

### 3.5. Mechanical Properties

**Figure 8:**
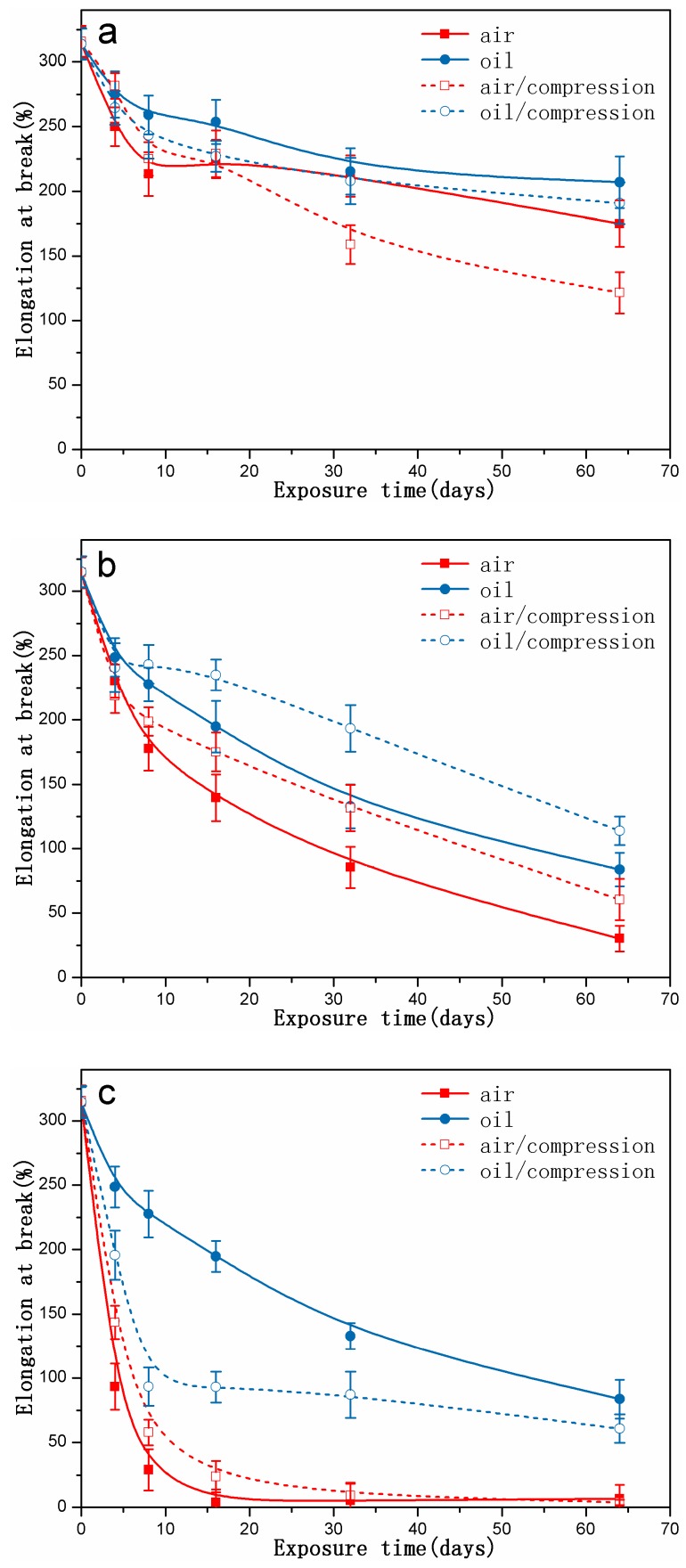
The changes in elongation at break of rubber specimens exposed to different conditions as a function of exposure time: (**a**) at 70 °C; (**b**) at 90 °C; (**c**) at 110 °C.

Figure 8 presents the elongation at break of rubber specimens exposed to different conditions with increasing exposure time. With the exposure time increase, for all the cases, the elongation at break was found to decrease. However, for the specimens aged at 70 °C and 110 °C, the value tended to rapidly plateau with increasing exposure time. For the specimens aged at 90 °C and 110 °C, we observed that the value for exposure to air or hydraulic oil under free was lower as compared to that under compression, and the value under compression or not under compression aged in hydraulic oil presented more of an increase than that aged in air.

**Figure 9:**
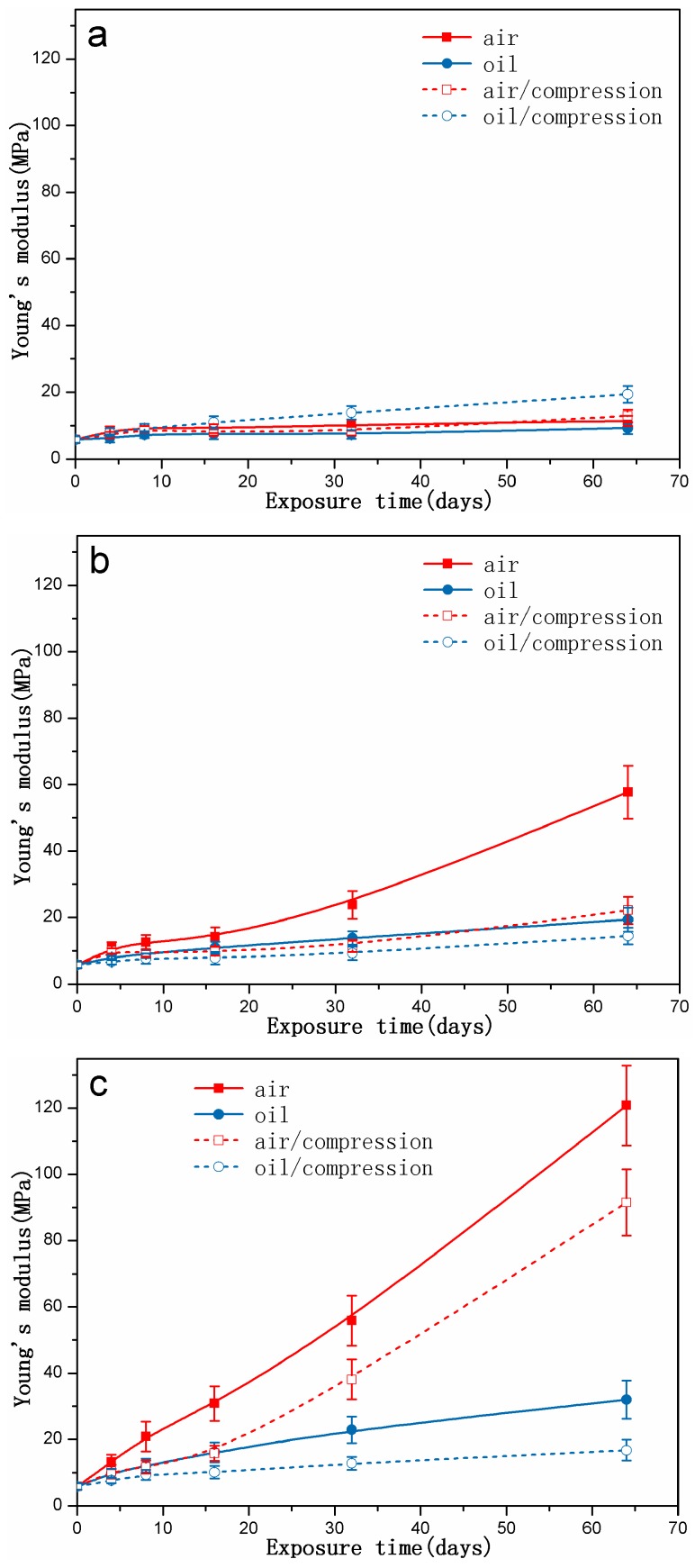
The changes in Young’s modulus of rubber specimens exposed to different conditions as a function of exposure time: (**a**) at 70 °C; (**b**) at 90 °C; (**c**) at 110 °C.

Figure 9 shows the changes in Young’s modulus of the rubber specimens exposed to different conditions with increasing exposure time. The Young’s modulus of all specimens continued to increase with increasing exposure time and temperature. Additionally, the Young’s modulus for the specimens stored under compressive stress was lower than that without stress under the same aging conditions. For most cases at 90 °C and 110 °C, a higher increase of Young’s modulus exposed to air was observed.

**Figure 10:**
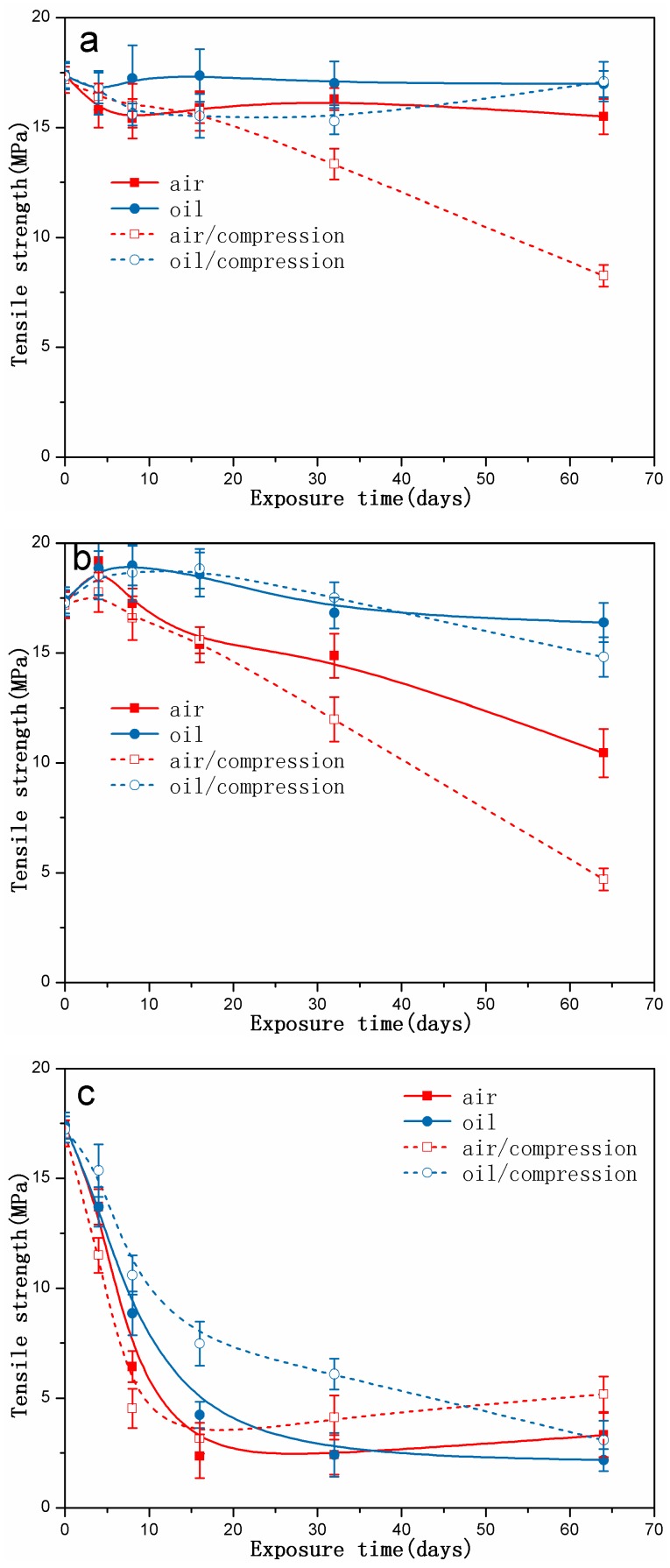
The changes in tensile strength of rubber specimens exposed to different conditions as a function of exposure time: (**a**) at 70 °C; (**b**) at 90 °C; (**c**) at 110 °C.

Figure 10 shows the tensile strength of rubber specimens exposed to different conditions as a function of exposure time. As shown in Figure 10a, the tensile strength of the compressed specimens exposed to air at 70 °C decreased slightly in the first 16 days, and then decreased markedly afterward. However, for the other cases, the tensile strength all changed little during the initial stage, but showed a slight increase in the later stage. When the rubber specimens were aged at 90 °C, as shown in Figure 10b, we found that the tensile strength all continued to increase during the first days and then decreased significantly, especially for the uncompressed and compressed specimens exposed to air. For the most exposure time, the tensile strength of the compressed specimens was lower than that of those under the free state under the same exposure conditions. Figure 10c presents the tensile strength of rubber specimens aged at 110 °C with increasing exposure time. It was observed that the changes of the tensile strength under different aging conditions presented a similar trend that the tensile strength decreased sharply at first and then remained constant afterward. In addition, the tensile strength of the uncompressed and compressed specimens exposed to hydraulic oil is higher than that of exposed to air, implying the more serious degradation for exposure to air.

On the basis of above results, it can be concluded that hydraulic oil, temperature, and compressive stress all have a direct influence on changes in mechanical properties, while the higher temperature has a stronger effect.

## 4. Discussion

The results of crosslinking density measurements after exposure to air clearly show that crosslinking and chain scission reactions both occurred simultaneously at higher temperature during the thermal aging process [5,7,20,21]. The competition between crosslinking and chain scission resulted in changes in crosslinking density [20,21]. As shown in Figure 2, for the specimens aged in air at 70 °C and 90 °C, crosslinking reactions dominated during the degradation process, leading to an increase of crosslinking density. Furthermore, the higher the temperature was, the faster the crosslinking was. However, for the specimens aged in air at 110 °C, crosslinking reactions were dominant at the initial stage, causing the rapid increase in crosslinking density, whereas the chain scission reactions predominated after that and inhibited the increase of crosslinking density. Additionally, the alkyl, alkoxy or peroxy radical could connect with each other to result in occurrence of crosslinking [7,41]. The results of ATR-FTIR measurements before and after exposure to air present that oxidation reactions also occurred during the degradation process, including the formation of oxygenated species like hydroxyl groups, carboxyl groups, and amide groups. Moreover, oxygenated species could also combine with each other to form further crosslinking [5,42]. This may well explain the complicated changes in intensity of the peak at 1735 cm^−1^ with exposure and high temperature. On the other hand, on the basis of the results of ATR-FTIR and SEM measurements, physicochemical changes also were observed. It could be concluded that the migration or/and volatilization of additives, the formation of voids, particle agglomerates, and hardened brittle outer layer occurred during the degradation process. The loss of additives such as antioxidants weakened the oxidation resistance of the rubber matrix, and thus generation of thermal oxidation was easier. The appearance of defects on the fracture surface demonstrate the poor compatibility between fillers and the rubber matrix. Additionally, the heavily oxidised layer also implies that the oxidation gradually extended from the surface to the internal structure. All these physical and chemical changes both have a significant effect on mechanical properties. The additives loss such as plasticizer resulted in a decrease of mechanical properties at the initial stage. Additionally, with increasing exposure time and temperature, the plasticizer and antioxidant loss and the increase in the crosslinking density made the network structure denser and denser, and rubber materials turned brittle. Formation of defects and the heavily oxidised layer were attributed to degradation in mechanical properties. These reasons could be well explained for the decrease in elongation at break and increase in Young’s modulus at the later stage. Obviously, the changes in tensile strength at 90 °C show a big difference as compared to that at 70 °C and 110 °C. This could be explained that the moderation crosslinking of vulcanized rubber formed a large amount of effective cross-link points with the increase of crosslink density and helped to disperse and transfer stress, which could cause the increase of the tensile strength at 90 °C during the first phase. However, for the higher crosslinking density, the crosslinking points were also more and limited the activities of the segments, which were not beneficial for the dispersion of stress, and thus led to decrease of the tensile strength [4,11,40]. Besides, the decrease of the tensile strength might partly be due to the loss of the additives at the earlier stage. Furthermore, at 110 °C, the chain scission reactions held the dominant position and destroyed seriously the rubber matrix structure, resulting in a decrease in tensile strength at the later stage.

When the specimens were immersed in hydraulic oil at high temperatures, the rubber tended to swell and/or degrade, which was mainly due to the solvent uptake and the relaxation of rubber chains. Swelling was attributed to the interaction between the material and solvent [25]. In addition, the hydraulic oil could dissolve or extract soluble components (e.g., antioxidants) from the rubber during the exposure process. These changes may result in poor oxidation resistance, and lead to changes in the mass and dimensions of rubber specimens, which may affect the mechanical properties, compression set, etc. [5,23,24]. As observed in Figure 2, it was found that the thermal aging effects on rubber network structures showed a similar trend. Both crosslinking and chain scission reactions also happened during the thermal aging process. Additionally, for all cases, the crosslinking density in hydraulic oil at three different temperatures were lower than that in air, implying the faster degradation rate for the rubber specimens in air. Nevertheless, the amide groups were formed after 32 days of exposure to hydraulic oil at 70 °C (shown in Figure 3), but not in air. At 110 °C, the peaks representing the amide groups for the specimens exposed to hydraulic oil showed the higher intensity. These phenomenon both indicated that the synergistic action of elevated temperature and hydraulic oil could promote the reactions of nitrile groups. On the other hand, the intensity of peaks at 2915 cm^−1^, 2852 cm^−1^, 1735 cm^−1^, 1577 cm^−1^, and 1179 cm^−1^ all presented more of a decrease in hydraulic oil than that in air. These demonstrated that hydraulic oil could extract oil-soluble additives [8,42], which resulted in more loss of additives as compared to that in air, which matched well with the more voids on the fracture surface especially at higher temperature. This led to the poorer resistance to oxidation of the matrix. The thicker oxidised layer on the specimen surface also indicated this. However, for the specimens immersed in hydraulic oil, hydraulic oil surrounded the specimens and limited significantly oxygen access in specimens, while a certain amount of oxygen still penetrated into the seals via diffusion [30,35]. Moreover, the antioxidant present in oil could probably reduce the amount of oxygen reaching the rubber surface. The limited oxygen for oil-immersion specimens compared to air-exposure specimens slowed the oxidation process in hydraulic oil, which was in agreement with the lower crosslinking density in oil. In addition, there is an excellent correlation between Young’s modulus and crosslink density [27,31]. Thus, the Young’s modulus in air is higher than that in hydraulic oil. Whereas, the double effect between more additives loss and physicochemical reactions in hydraulic oil at higher temperature resulted in more of a decrease of elongation at break and tensile strength.

When rubber seals were subjected to compression stress to keep a constant deformation at elevated temperature, according to results of crosslinking density measurements, the degradation of rubber seals still occurred via crosslinking reactions and chain scission reactions at elevated temperature, whereas crosslinking predominated overall the degradation process at three temperatures, and thus resulted in the sustained increase of crosslinking density during the degradation process. Besides, at the first phase, the compression stress could break rubber molecular chains, which produced free radicals, namely stress-induced chain scission. Thus, the chain scissions mainly consisted of stress-induced and oxidation-induced chain scission. This might explain the fact that the increase rate of crosslinking density under compression at 90 °C and 110 °C was slower than that under the free state at the initial stage. Afterward, the free radicals were oxidized under the combined action of heat and oxygen, leading to additional oxidation or/and crosslinking [7,35]. This could explain the phenomenon that the increase rate in crosslinking density under compression over time at 70 °C, 90 °C, and 110 °C after middle stage was faster than that under the free state in air or hydraulic oil. However, in order to keep the constant deformation of rubber seals, we designed the compression apparatus to simulate the actual compression state. The compression apparatus could inhibit the oxygen access in the rubber seals, which could not catch up with the oxygen consumption during the aging process. Due to the shortage of oxygen, the aging degree of rubber seals was lower than that in air under the free state at 90 °C. Compared to crosslinking density under compression at 90 °C, the lower value in hydraulic oil after 64 days of immersion indicated that the hydraulic oil effect on limiting the oxygen access was greater than the compression stress. At the same time, the stress-induced physical changes also occurred with chemical changes. The compression stress could promote the rearrangement of rubber chains and fillers and the orientation of molecular chains, which restricted the movement of the molecular chains or fillers and slowed the penetration of gas-liquid molecules into the rubber interior. The less decrease in intensity at peaks at 2915 cm^−1^, 2852 cm^−1^, 1735 cm^−1^, and 1577 cm^−1^ implied that the loss of additives was lower than that under the free state. These behaviors took place within a very short time after deformation of the rubber seals. By comparison of the typical spectra for the uncompressed and compressed samples aged at 70 °C and 110 °C, no formation of amide groups was observed over time for the aged samples under compression. Additionally, the intensity of the peak at 2232 cm^−1^ under compression further indicated that the reactions of nitrile groups did not generate during the whole period. The changes in intensity of the peak at 967 cm^−1^ demonstrated that the rubber seals aged without stress showed more serious degradation than that with stress. The fracture surface of the compressed samples after 32 days of exposure showed the lesser defects and a thinner oxidized layer on the specimen surface than that in air and hydraulic oil under the free state. This phenomenon could be explained that the compressive stress limited the diffusion of oxygen into the rubber interior and slowed the degradation process. The results of compression set show that the compression set increased with increasing exposure time and temperature. This was mainly due to loss of additives, crosslinking, and chain scission (stress-induced and oxidation-induced). All these physical and chemical changes degraded the elongation at break and Young’s modulus. At higher temperature, the value in air under compression was lower than that in air under free, but higher than that in hydraulic oil under free. However, more serious destruction of network structure and restriction of rubber chains were not beneficial for dispersal and transfer of stress, leading to more of a decrease than that of aged in air not under compression.

When specimens were subjected to the synergistic effects of elevated temperature, hydraulic oil and compressive stress simultaneously, the chemical changes presented the similarity with that aged in air under compression for most cases. The crosslinking reactions and chain scission reactions (stress-induced and oxidation-induced) competed with each other, resulting in the complicated changes of crosslinking density. For the specimens aged at 70 °C and 90 °C, the crosslinking reactions were believed to dominate during the whole degradation process, causing the increase of crosslinking density. However, at 110 °C, the crosslinking reactions dominated at the initial stage, but chain scission reactions predominated after eight days of exposure, leading to the decrease in crosslinking density. Furthermore, rubber seals immersed in hydraulic oil under compression at three temperatures all showed the minimum degradation level as compared to that aged under the other conditions. On the basis of the results of ATR-FTIR, we found that the complicated changes in peak intensity at 1735 cm^−1^ and 1577 cm^−1^ under different exposure conditions well indicated that compression stress could greatly restrict the migrations of additives, and hydraulic oil could well extract the oil-soluble additives. Besides, compression stress and hydraulic oil both could limit the oxygen access in rubber interior and retard the thermal aging. Furthermore, the effects of oil limiting the diffusion of oxygen showed more pronounced than the effects from compressive stress. Compression stress also inhibited the hydraulic oil to penetrate into rubber seals, which weakened indirectly the effect of hydraulic oil. The synergistic effect between compression stress and hydraulic oil on further slowing the thermal aging was obviously observed from the changes in crosslinking density under different aging conditions. The formation of defects on the fracture surface of specimens aged in hydraulic oil under compression were mainly attributed to stress-induced the rearrangement of rubber chains and swelling or/and extraction of hydraulic oil at elevated temperature. These might explain the fact that the mechanical properties of compressed specimens exposed to oil at higher temperatures presented the minimum degradation level as compared to that aged under the other aging conditions.

## 5. Conclusions

In this work, the synergistic effects of elevated temperature, hydraulic oil, and compressive stress on degradation behaviors and mechanisms of hydrogenated nitrile rubber seals were investigated. The results obtained from crosslinking density show that the rubber materials degraded via the competition between crosslinking and chain scission, and for most cases, the crosslinking reactions predominated during the degradation process. Meanwhile, the higher temperature and compressive stress promoted the occurrence of oxidation-induced and stress-induced chain scission, respectively. Additionally, the synergistic effect between compression stress and hydraulic oil further slowed the degradation process by restraining oxygen access in the rubber interior. The ATR-FTIR results indicate that the higher temperature and hydraulic oil promoted the formation of oxidation products, especially under synergistic effects of these two factors, whereas the reactions of nitrile groups did not generate for the compressed specimens. The fracture morphology results show that the defects gradually formed on the fracture surface due to oxidation reactions, loss of additives, and swelling of hydraulic oil, especially for the uncompressed specimens.

In terms of physical and mechanical properties of rubber materials, the compression set and Young’s modulus increased, and the elongation at break decreased with increasing exposure time and temperature. However, tensile strength shows a bigger difference at different temperatures. These changes in physical and mechanical properties are mainly attributed to the physical and chemical processes, such as the loss of additives, degradation, and swelling of network structure, formation of defects, and the rearrangement of rubber chains, etc. The results imply that the compressed rubber seals exposed to hydraulic oil presented the minimum degradation level. The degradation of specimens exposed to hydraulic oil is more serious than that of specimens exposed to air. The compression stress accelerated the degradation of the mechanical properties at low temperature, but restrained at higher temperature.

## Figures and Tables

**Table 1:** Constituents of the hydrogenated nitrile butadiene rubber (HNBR) samples.

Constituents	Phr (Parts per Hundreds of Rubber)
Rubber	100
Zinc oxide	5
Sulfur	2.5
Carbon black	50
Plasticizer	18
Stearic acid	1.5
Antioxidant	2

**Table 2:** Peak assignments.

Wavenumber (cm^−1^)	Peak Assignment	References
2915	–C–H stretching vibration in the CH_2_ group	[5,10,11,21]
2852	–C–H stretching vibration in the CH_2_ group	[5,10,11,21]
2233	–CN stretching vibration	[5,10,11,21]
1735	–C=O stretching vibration	[5,10,11,21]
1577	C–O–C stretching vibration	[5,10,18,20,21]
1432	–C–H bending vibration in the CH_2_ group	[5,10,11,21]
1179	additives such as stearic acid and plasticizer	[5,10,18,20,21]
967	–C–H deformation vibration for 1,4-trans-vinylene	[5,10,11,18,21]
